# Ammonium Uptake by Phytoplankton Regulates Nitrification in the Sunlit Ocean

**DOI:** 10.1371/journal.pone.0108173

**Published:** 2014-09-24

**Authors:** Jason M. Smith, Francisco P. Chavez, Christopher A. Francis

**Affiliations:** 1 Research Division, Monterey Bay Aquarium Research Institute, Moss Landing, California, United States of America; 2 Department of Environmental Earth System Science, Stanford University, Stanford, California, United States of America; Laval University, Canada

## Abstract

Nitrification, the microbial oxidation of ammonium to nitrate, is a central part of the nitrogen cycle. In the ocean’s surface layer, the process alters the distribution of inorganic nitrogen species available to phytoplankton and produces nitrous oxide. A widely held idea among oceanographers is that nitrification is inhibited by light in the ocean. However, recent evidence that the primary organisms involved in nitrification, the ammonia-oxidizing archaea (AOA), are present and active throughout the surface ocean has challenged this idea. Here we show, through field experiments coupling molecular genetic and biogeochemical approaches, that competition for ammonium with phytoplankton is the strongest regulator of nitrification in the photic zone. During multiday experiments at high irradiance a single ecotype of AOA remained active in the presence of rapidly growing phytoplankton. Over the course of this three day experiment, variability in the intensity of competition with phytoplankton caused nitrification rates to decline from those typical of the lower photic zone (60 nmol L^−1^ d^−1^) to those in well-lit layers (<1 nmol L^−1^ d^−1^). During another set of experiments, nitrification rates exhibited a diel periodicity throughout much of the photic zone, with the highest rates occurring at night when competition with phytoplankton is lowest. Together, the results of our experiments indicate that nitrification rates in the photic zone are more strongly regulated by competition with phytoplankton for ammonium than they are by light itself. This finding advances our ability to model the impact of nitrification on estimates of new primary production, and emphasizes the need to more strongly consider the effects of organismal interactions on nutrient standing stocks and biogeochemical cycling in the surface of the ocean.

## Introduction

The quantity of nitrogen (N) supplied to the sunlit layers of the ocean regulates levels of primary production and phytoplankton community composition [1]. The general assumption is that nitrate (NO_3^−^_) entering the photic zone from deeper layers serves as the additional source of N needed to support ‘new’ primary production, and therefore the traditional measurement of new production has been NO_3^−^_ uptake by phytoplankton [2]. Furthermore, the vertical flux of carbon has been assumed to be equal to new production over the appropriate time and space scales [1]. The occurrence of nitrification in the photic zone complicates these paradigms by providing a regenerated source of NO_3^−^_. Accounting for this process is therefore needed in order to make accurate estimates of new primary production [2], and the strength of the ocean’s biological pump [1].

Despite decades of observations of nitrification in the photic zone [3]–[8], the impacts of this process on global estimates of new production were assessed only recently. From this recent meta analysis, it was suggested that between 18 and 33% of NO_3^−^_ in the photic zone is regenerated within it by nitrification, causing model-based estimates of oceanic new production to be 1.5 to 3-fold higher than actual [9]. The primary sources of uncertainty in these estimates are the poor spatiotemporal coverage in the global data set [9], and the fact that we have yet to establish strong relationships between ecological and environmental factors and nitrification.

It has long been believed that nitrification is regulated by light in the photic zone of the ocean. Primary support for this hypothesis comes from repeated reports of nitrification rates being low in the surface mixed layer and then increasing exponentially with depth, as irradiance intensity decreases, to a maximum near the photosynthetic light compensation point (1% blue light) [3]–[8]. Prior to the discovery of the ammonia-oxidizing archaea (AOA) [10], the light inhibition hypothesis was also bolstered by experimental results showing some marine ammonia-oxidizing bacteria (AOB) to be light sensitive [11]–[15]. However, AOB are typically absent or present at much lower abundances than AOA in the photic zone [16], [17]. Therefore, any sensitivity of nitrification to light would be due to inhibition of the AOA [17], [18]. Growth inhibition by light in cultures of AOA isolated from soils and sediments was recently reported [19], [20]. Whether these findings are upheld by AOA in the ocean remains to be determined. Numerous observations of nitrification in the photic zone [4]–[7], [21], and recent reports that AOA are present and expressing the gene products (i.e., *amoA* mRNA transcripts) required to carry out this process [18], [22]–[24], suggest that nitrification is not inhibited by light in the photic zone of the ocean.

As the ultimate driver of photosynthesis, light intensity in a given ocean layer influences rates of primary production as well as the rate that N, a widely limiting and essential macronutrient, is withdrawn from seawater by phytoplankton [25]. The N demand of phytoplankton, which also co-varies with depth in the water column, creates varying intensities of competition, primarily for ammonium (NH_4^+^_) [2], [25]. We hypothesize that competition with phytoplankton for NH_4^+^_ plays a larger role than light itself in determining the distribution of nitrification rates and activity of the AOA in the photic zone of the ocean.

Isolating the influence of light or competition on nitrification remains difficult, because their intensities co-vary in stratified surface waters [5], [25], [26]. Therefore, results of experiments with surface waters may indicate a direct effect of irradiance intensity on rates of nitrification when, in fact, the experimental conditions altered phytoplankton growth rates and the degree of competition for NH_4^+^_. In upwelling systems, disphotic waters, high in nutrients and free of growing phytoplankton, are transported to the surface layer where they are first exposed to light and then, a few days later, rapidly growing phytoplankton [27], [28]. To test our competition hypothesis, experiments mimicking the upwelling and maturation of a water mass were performed, with the goal of isolating the effects of light and phytoplankton growth on the activity of ammonia-oxidizing microorganisms and rates of nitrification.

## Results and Discussion

### Direct effects of light on nitrification

To isolate the effects of light on natural ammonia-oxidizing microbial communities, rates of nitrification in waters from depths with low or no phytoplankton biomass (Table 1) were determined following incubation (24 h) in continuous darkness or exposed to the irradiance and photoperiod of the mixed layer (Fig. S1). The detection of ammonia monooxygenase (*amoA*) genes and mRNA transcripts associated with three major groups of marine ammonia oxidizing microorganisms (AOM), ‘Water Column A’ (WCA) and ‘Water Column B’ (WCB) ecotypes of the AOA and betaproteobacterial ammonia oxidizing bacteria (β-AOB), confirmed that putatively light-sensitive AOM were present and active at the start of our experiments (Table 1). These data also revealed that the WCA ecotype of AOA comprised the majority of *amoA* gene (79%±20%) and transcript (88%±5%) pools in all samples (Table 1).

**Table 1:** Physicochemical and biological properties of waters used for determining the effects of light on rates of nitrification in the absence of rapidly growing phytoplankton.

	Station M1		Station M2		
	30 m	60 m	30 m	60 m	200 m
Pressure	30.2	60.5	31.5	61.4	202
NO_3^−^_+NO_2^−^_ (µM)	12.1	18.9	5.1	19.6	30.6
Chlorophyll (µg L^−1^)	0.8	0.1	1.7	B.D.†	B.D.
PAR (% of Surface)	1.5%	0.1%	6%	0.1%	<0.1%
WCA *amoA* (genes L^−1^)††	4×10^6^	4×10^6^	1×10^6^	6×10^6^	6×10^6^
WCB *amoA* (genes L^−1^)	9×10^4^	2×10^5^	9×10^3^	3×10^4^	4×10^6^
AOB *amoA* (genes L^−1^)	2×10^5^	3×10^5^	9×10^4^	5×10^4^	2×10^4^
WCA *amoA* (mRNA L^−1^)	4×10^5^	3×10^5^	6×10^4^	2×10^6^	1×10^6^
WCB *amoA* (mRNA L^−1^)	2×10^3^	5×10^3^	B.D.†	3×10^4^	3×10^5^
AOB *amoA* (mRNA L^−1^)	8×10^4^	2×10^4^	6×10^3^	5×10^4^	B.D.

† B.D, below detection (<0.01 µg L^−1^ chlorophyll or <10 copies per qPCR reaction for AOB *amoA*).

†† Gene and transcript abundances obtained by qPCR were normalized to the volume of seawater filtered.

Given laboratory evidence that AOA isolates are unable to resume growth following light exposure [19], [20], we expected to observe substantially lower rates of nitrification in disphotic waters subject to 12 h of surface irradiance. However, no statistically significant trend in support of this hypothesis was found (t-test, P>0.05). Differences in rates averaged 15% (±7%, S.D.), but were not consistent between treatments (Table 2). Based on these experiments, we conclude that light exposure does not have a prolonged inhibitory effect on pelagic AOA activity or nitrification rates.

**Table 2:** Results of light inhibition experiments, when waters from dark or dimly lit depths, where phytoplankton growth was minimal, were exposed to the irradiance and photoperiod of the surface layer or held in complete darkness for 24 h.

Station	Depth (m)	Nitrification (nmol L^−1^ d^−1^)	
		50% light	Dark
M1	30	93.9±1.5†	83.7±2.9
	60	94.5±0.4	106.2±0.2
M2	30	14.8±0.6	19.5±1.7
	60	86.3±2.9	77.2±7.0
	200	14.7±0.2	11.2±1.3

† Values represent the mean ± standard deviation of duplicate samples at each depth (N = 2).

### Response of nitrification to variable rates of phytoplankton growth

The effects of phytoplankton growth on nitrification and AOM were studied using multiday experiments with three water types common to upwelling environments, namely: surface waters with actively growing phytoplankton (designated ‘surface’), comprised mainly of centric diatoms (42% of biomass) (Table S1); low chlorophyll, deep waters brought to the surface (designated ‘deep’); and the same deep waters seeded with surface waters (designated ‘bloom’), to induce a phytoplankton bloom [29]. All treatments were incubated for three days under the irradiance (1300 to 1800 µE m^−2^ s^−1^) and photoperiod (12 h) of the surface layer (Fig. S1).

**Figure 1:**
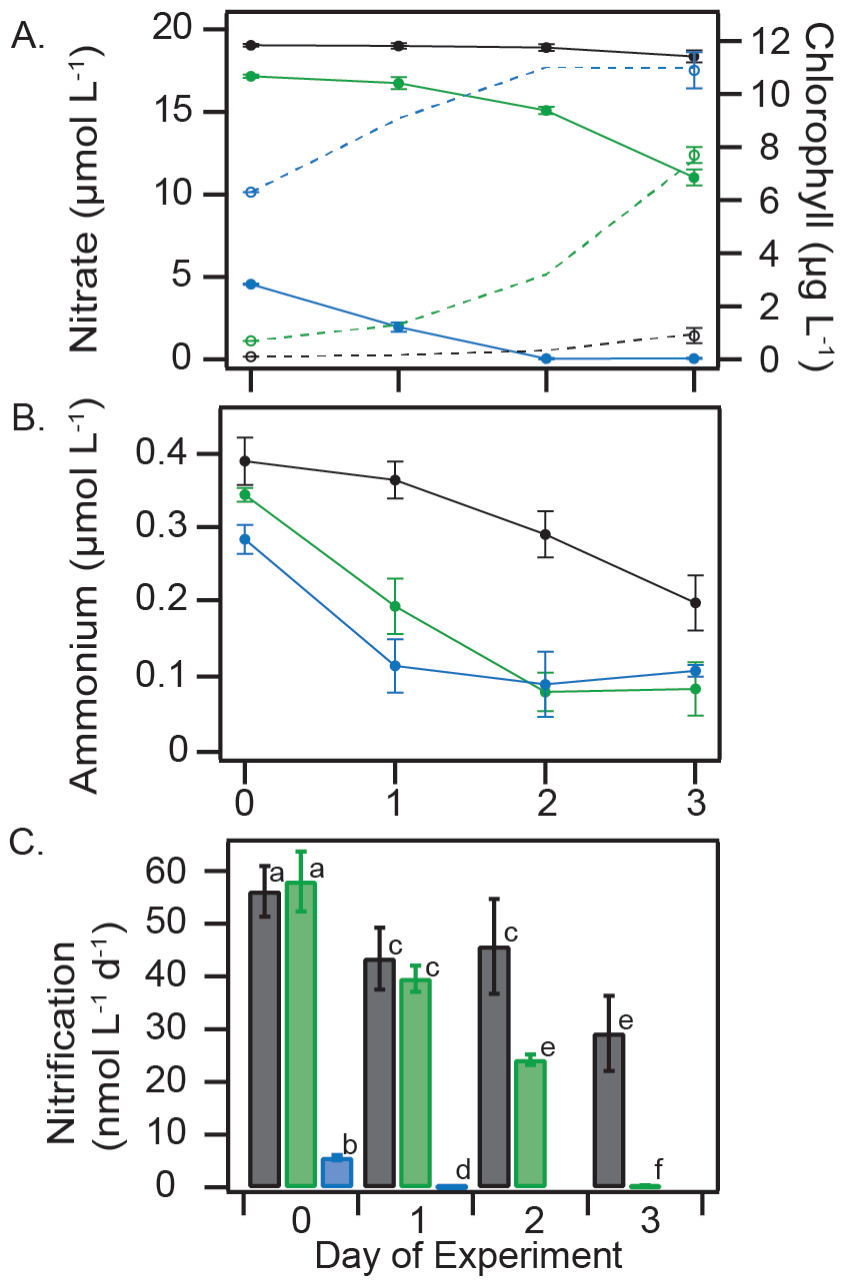
Results of multiday competition experiment showing the time-course of NO_3^−^_, chlorophyll and NH_4^+^_ concentrations and nitrification rates. (A) Concentrations of NO_3^−^_ (solid lines) and chlorophyll (hashed lines) in ‘surface’ (blue), ‘deep’ (black) and ‘bloom’ (green) treatments; (B) NH_4^+^_ concentrations; (C) and nitrification rates. Error bars (standard deviations of triplicate samples for all data except nitrification rates in ‘deep’ which are duplicates) are included on all data points, but in many cases are smaller than the symbols. Letters in panel (C) denote statistically supported differences in groups of means (t-test, P<0.05), the same letter on separate bars denotes rates that were not statistically different.

By the end of our experiments, phytoplankton uptake consumed 4.5, 0.7, and 6.1 µmol L^−1^ of NO_3^−^_ in ‘surface’, ‘deep’ and ‘bloom’ treatments, respectively (Fig. 1A), which corresponded to increases in chlorophyll from 6.3 to 10.9 µg L^−1^ in ‘surface’, 0.1 to 1.7 µg L^−1^ in ‘deep’ and 0.7 to 7.8 µg L^−1^ in ‘bloom’ (Fig. 1A). NH_4^+^_ concentrations decreased from 0.28 to 0.11, 0.39 to 0.2 and 0.34 to 0.08 µmol L^−1^, in ‘surface’, ‘deep’ and ‘bloom’ treatments (Fig. 1B). Nitrification rates decreased from 5.6 to 0.2 nmol L^−1^ d^−1^ (4% of initial) by Day 1 in ‘surface’, after which no activity was detected. The ‘deep’ and ‘bloom’ had remarkably similar initial rates of 57 and 58 nmol L^−1^ d^−1^. By the end, rates declined to 29 nmol L^−1^ d^−1^ (50% of initial) in ‘deep’ and to 0.3 nmol L^−1^ d^−1^ (0.5% of initial) in ‘bloom’ (Fig. 1C).

**Figure 2:**
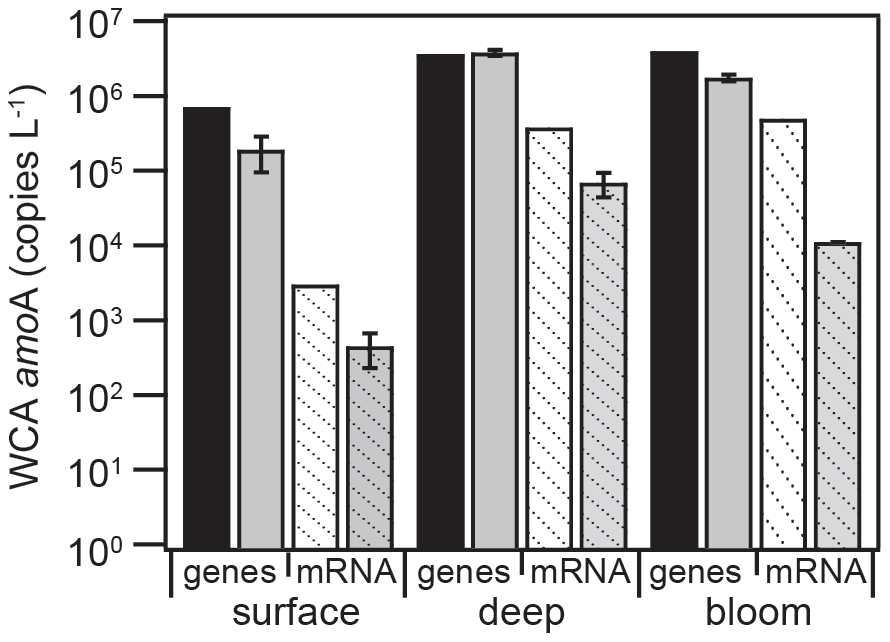
The abundance of WCA AOA *amoA* genes (solid bars) and mRNA transcripts (hashed bars) at the beginning (black for DNA, white for RNA) and end (gray) of the multiday experiments. Initial time points from the start of the experiment were not replicated. End time points, taken on Day 3, are presented with error bars representing the standard deviation about the mean of triplicate (‘surface’ or ‘bloom’) or duplicate (‘deep’) samples. Gene and transcript abundances obtained by qPCR were normalized to the volume of seawater filtered.

Assessment of *amoA* gene abundances at the start of the experiment indicated an AOM community comprised of 87–97% WCA, 0.5–5% WCB, and 3–8% β-AOB (Table S2). WCB and β-AOB *amoA* genes were present in all end point samples. However, their *amoA* mRNA transcripts were not detected at all in ‘surface’ or on Day 3 in ‘bloom’ and ‘deep’ (Table S2), preventing us from determining whether light or phytoplankton growth inhibited their activity. WCA *amoA* genes and transcripts were present in all treatments and time points. The declines in WCA *amoA* transcripts consistently surpassed those observed for their genes, by 7- vs. 4-fold in ‘surface’, 5-fold vs. unchanged in ‘deep’, and 45- vs. 2-fold in ‘bloom’ (Fig. 2).

Overall, these data indicate that there was no net growth of the WCA or WCB AOA or the AOB (Table S2), during the three-day experiments (Fig. 2). A doubling time of more than 3 days is consistent with 4 to 4.5 day doubling times observed in laboratory enrichments of WCA AOA, started from seawater collected in the same region of the northeast Pacific Ocean [30]. Instead, WCA a*moA* mRNA abundances showed much larger relative declines over the course of our experiments, which suggests that phytoplankton growth had a stronger effect on WCA AOA cellular activity than it did on their community size (Fig. 2). We assert that the observed changes in WCA AOA cellular activity and rates of nitrification were due to the inability of the AOA to effectively compete for NH_4^+^_ with phytoplankton.

Nitrification [31]–[33] and the uptake of NH_4^+^_ by phytoplankton [34]–[38] exhibit patterns of substrate uptake that conform to the Michaelis-Menten model. In other words, the outcome of competition between them is largely determined by two factors: the maximum rate of uptake (V_max_) for the shared substrate and the half-saturation value (K_m_), or the substrate concentration supporting half the maximum rate of nutrient uptake [39]. The question as to which of the two kinetic traits, V_max_ or K_m_, was most important in determining the outcome of our experiments can be addressed by considering the results of the multiday enclosures (Fig. 1A-B; Fig. 2) and ^15^N-NH_4^+^_ tracer incubations as separate (Fig. 1C) but complimentary experiments.

NH_4^+^_ concentrations in the enclosures, to which no ^15^N tracer was added, reached a baseline of approximately 0.1 µmol L^−1^ in ‘surface’ and ‘bloom’ treatments (Fig. 1B), concurrent with the accumulation of chlorophyll (Fig. 1A). Once NH_4^+^_ reached this baseline, chlorophyll continued to accumulate and NO_3^−^_ showed distinct patterns of depletion (Fig. 1A). This observation suggests that once NH_4^+^_ concentrations were depleted to this baseline of approximately 0.1 µmol L^−1^, NH_4^+^_ uptake by phytoplankton became substrate-limited and the community began relying on nitrate to continue its growth (Fig. 1A-B). To support its growth, the AOA community could have used the sizeable pool of residual NH_4^+^_. However, there were no clear signs of further NH_4^+^_ depletion, or increased cellular activity of the AOA (Fig. 2), once NO_3^−^_ became the dominant N source supporting phytoplankton growth (Fig. 1A-B).

Prior experiments performed in the Sargasso Sea [33] and Puget Sound [32] determined the K_m_ of pelagic ammonia oxidation to range from 0.07–0.15 µmol L^−1^, similar to that of *Nitrosopumilus maritimus* SCM1 (0.134 µmol L^−1^), isolated from aquarium sediments [31]. We observed a strikingly similar baseline NH_4^+^_ concentration in both ‘surface’ and ‘bloom’ treatments (Fig. 1B). However, when the ^15^N-based rate data are used to estimate the contribution of AOA to NH_4^+^_ depletion in the enclosures, the results suggest that only 2% (0.0056 of 0.28 µmol L^−1^), 15% (0.057 of 0.39 µmol L^−1^) and 17% (0.058 of 0.34 µmol L^−1^) of the substrate pool was oxidized during the first day of the experiment in ‘surface’, ‘deep’ and ‘bloom’ (Fig. 1B-C). Over the course of the experiment, these contributions decreased, along with AOA cellular activity (Fig. 2). This means that nitrification was the not the primary cause of the NH_4^+^_ depletion in the enclosures. Therefore, the baseline NH_4^+^_ concentration of approximately 0.1 µmol L^−1^ observed in ‘surface’ and ‘bloom’ reflects the concentration at which NH_4^+^_ uptake by phytoplankton becomes substrate limited. The fact that this concentration happens to be similar to the K_m_ for pelagic ammonia oxidation suggests two things. First, both phytoplankton uptake of NH_4^+^_ and nitrification are substrate limited, which means that they are in direct competition for NH_4^+^_ in the well-lit layers of the photic zone. Second, the AOA are able to persist during short periods of starvation (Fig. 2). In order to explain changes in the distribution of their biomass in the surface ocean, a factor other than competition for NH_4^+^_, such as mixing, grazing or viral lysis, has to be invoked.

**Figure 3:**
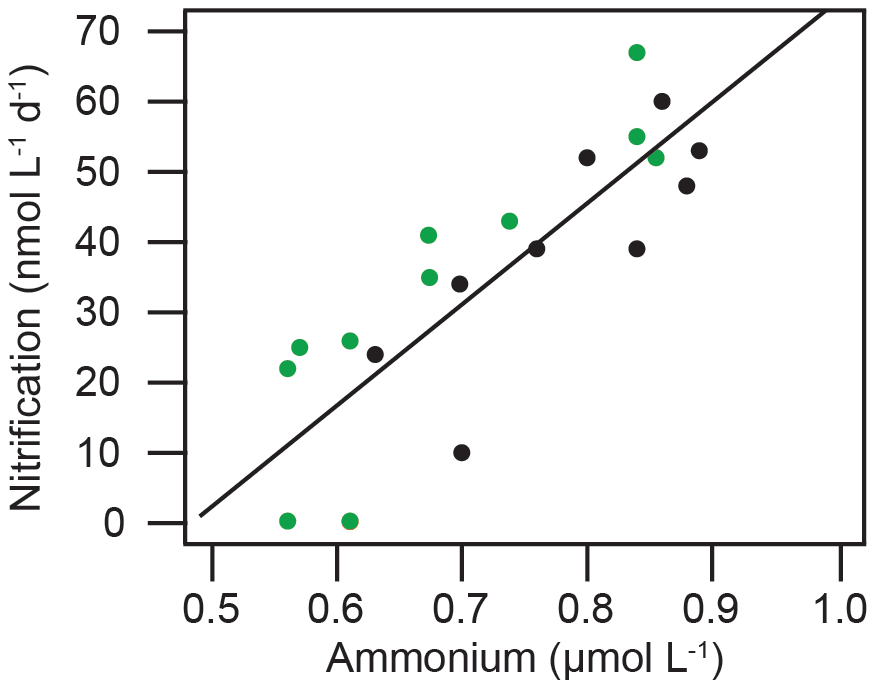
Regression plot showing the linear relationship between nitrification rates and ammonium concentrations (ambient + ^15^N tracer) in the ‘deep’ (black) and ‘bloom’ (green) treatments during multiday experiments. Data from ‘surface’ are not included because rates were no longer detectable after Day 1 of the experiment. The fit was found to be significant (P<0.01, R^2^ = 0.72).

Interpreted in the context of the Michaelis-Menten model, the linearity of the rate-substrate relationship in our dataset (Fig. 3) shows no signs of nitrification becoming substrate saturated in the photic zone. It further suggests the K_m_ of ammonia oxidation in the photic zone to be substantially higher than has been determined at depths below it (in the absence of phytoplankton) [32], [33]. These results could be interpreted as evidence that the kinetic traits of pelagic AOA are widely variable. More likely, they indicate that there is an additional factor, aside from the flux of NH_4^+^_ from remineralization, that influences nitrification rates in the photic zone.

The substrate-rate relationship (Fig. 3) was determined in experiments where both NH_4^+^_ concentrations and phytoplankton abundances varied, meaning the data are not the result of a classic kinetics experiment [32], [33]. However, the AOA are purportedly able to maintain their growth at NH_4^+^_ concentrations <10 nmol L^−1^. If the AOA have complete command of their substrate pool, the basic tenets of the kinetic model still hold true, whether phytoplankton are present or not. In our results, this would be indicated by nitrification rates increasing with substrate concentrations to asymptotically approach a maximum rate, as has been observed at much lower substrate concentrations in dark kinetics experiments [32], [33]. Or, as a constant rate of nitrification in the ‘deep’ treatment (Fig. 1C), when: chlorophyll accumulation and rates of NO_3^−^_ depletion were lowest (Fig. 1A), nitrification was not substrate limited (Fig. 1B), and the size of the active AOA community remained unchanged (Fig. 2). Yet, concentrations of WCA AOA *amoA* mRNA transcripts (Fig. 2) and rates of nitrification (Fig. 1C) decreased substantially in the ‘deep’ treatment, over a period of three days. We assert that this because phytoplankton are able to sequester NH_4^+^_ more rapidly than the slow growing AOA when it becomes available. Therefore, the upper boundary for nitrification rates in the photic zone is not set by the flux of NH_4^+^_ from remineralization, but by the size of the primary sink for it (phytoplankton). Once the primary sink is saturated, the residual NH_4^+^_ becomes available for nitrification.

**Figure 4:**
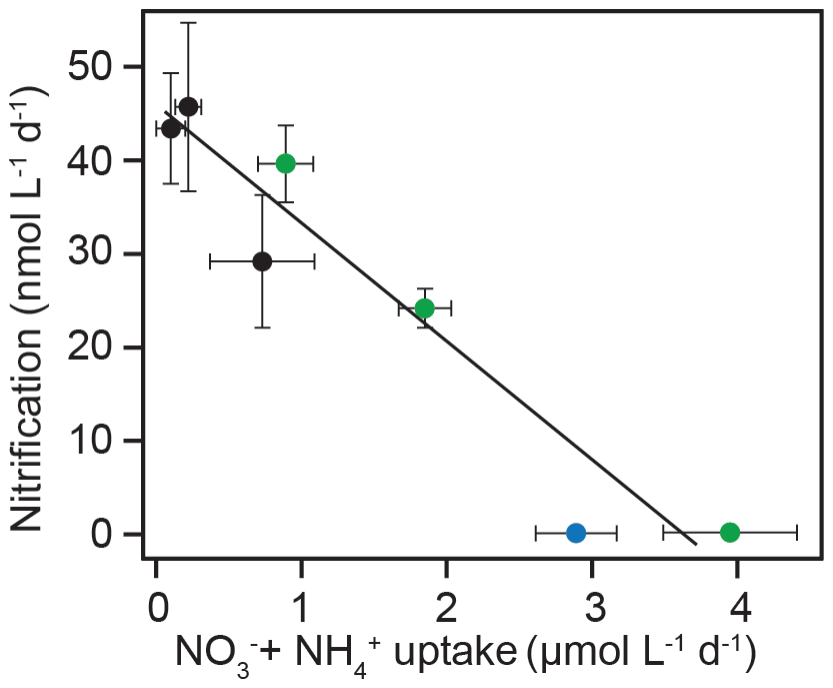
Regression plot of NH_4^+^_+NO_3^−^_ uptake rates, ascertained from differences between incubation days (enclosures) and nitrification rates on days 1–3 in ‘deep’ (black) and ‘bloom’ (green) and on Day 1 in ‘surface’ (blue), because rates were undetectable thereafter. Error bars represent the standard deviation about the mean of independent and dependent variables. The line of best fit has a slope (±S.D.) of −12.7±1.7 and intercept (±S.D.) of 45.3±3.5. The fit was found to be significant (P<0.01; R^2^ = 0.93).

Data from all treatments support an inverse-linear relationship (R^2^ = 0.93, P<0.01) between rates of nitrification and N depletion by phytoplankton, calculated as changes in concentrations of NH_4^+^_+NO_3^−^_ between days (Fig. 4). While it may seem counterintuitive to include NO_3^−^_ in the calculated uptake rates, the relationship works because diatom-dominated coastal phytoplankton assemblages (Table S1) maintain an equal, if not higher, V_max_ for NH_4^+^_ when growing with NO_3^−^_ [40]. Since NH_4^+^_ is the preferred substrate [40], the depletion rate of NO_3^−^_ once NH_4^+^_ concentrations reached a baseline (Fig. 1B) acts as a proxy for the total N demand of the community. The phytoplankton community consumed the least amount of NH_4^+^_ at the beginning of the experiment when growth rates, and N demand, were lowest. As the experiments progressed, increasingly more NH_4^+^_ went to support the demands of the growing phytoplankton community (with a higher V_max_), leaving incrementally less NH_4^+^_ available for nitrification (Fig. 4).

### Diel periodicity of nitrification rates in the photic zone

If competition for NH_4^+^_ with phytoplankton is the primary factor influencing nitrification rates in the photic zone, a nighttime increase in rates, when phytoplankton demand for NH_4^+^_ decreases [2], should occur. This idea was tested on the same cruise (further offshore, at station 67–70), by incubating waters from different depths in the photic zone at in situ light or in darkness for 24 hours.

**Figure 5:**
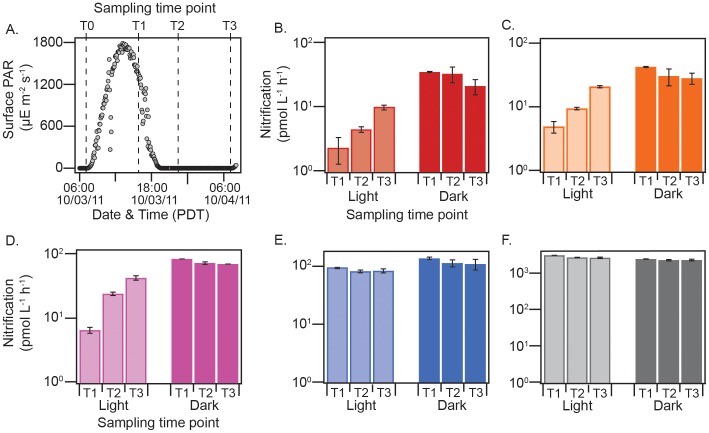
Results of diel periodicity experiments showing nitrification rates from five depths in the photic zone at station 67–70, incubated under in situ light conditions or in complete darkness for 24 h. (A) Surface photosynthetically active radiation (sPAR) during the experiment, with sampling intervals denoted by vertical hashed lines; (B) rates of nitrification at each sampling interval in surface waters (2 m) held at 90% sPAR or in the darkness; (C) from 5 m and held at 50% sPAR or in the dark; (D) from 10 m and held at 15% sPAR or in the dark; (E) from 20 m and held at 1% sPAR or in the dark; (F) and from 35 m and held at 0.1% sPAR or in the dark. Incubations were conducted in October of 2011 at station 67–70 in the Northeast Pacific. Error bars denote the standard deviation about the mean of duplicate samples.

At the time of sampling, there was a 30 m deep mixed layer, causing much of the photic zone to be well mixed. This resulted in ambient conditions at the start of the incubations being very similar between samples from 2, 5, 10 and 20 m, corresponding to 90, 50, 15 and 1% of surface irradiance (Fig. 5). Between these depths, temperature and salinity ranged from 15.51 to 15.52°C and 33.31 to 33.32, respectively. Chlorophyll, NH_4^+^_ and NO_3^−^_ concentrations ranged from 0.69 to 0.72 µg L^−1^, 2.6 to 3.8 µmol L^−1^ and 0.05 to 0.06 µmol L^−1^. Below the mixed layer, at 35 m, the water had a temperature and salinity of 11.2°C and 33.5, and contained 1.2 µg L^−1^ of chlorophyll, 16.5 µmol L^−1^ of NO_3^−^_ and 0.04 µmol L^−1^ of NH_4^+^_.

Nitrification rates were, again, detected during periods of high irradiance during these experiments. However, they continued to increase incrementally as irradiance decreased, reaching a maximum at night (Fig. 5A-B). A diel periodicity in nitrification rates was apparent at three irradiance intensities (90, 50 and 15% of surface irradiance) above the 1% isolume (Fig. 5B-D), below which light effects were not observed (Fig. 5E-F). In contrast, rates were strikingly consistent between time points when the same waters were held in darkness, which indicates that AOA activity and nitrification rates remain constant over a 24 h period in the absence of phytoplankton growth. Similar starting substrate and phytoplankton concentrations in samples from 2, 5, 10 and 20 m suggest that differences in the results between depths are due to the different irradiance intensities at which they were incubated (90, 50, 15 and 1% light, respectively).

Interpreted in the framework of our competition hypothesis, the results of our diel experiments support the idea that the N demand of phytoplankton increases along with irradiance intensity [2], [5], [25]. As a result, more of the daytime flux of NH_4^+^_ is consumed by phytoplankton at high irradiance, leaving less to be oxidized during the day (Fig. 5B). As irradiance decreases, so do the growth rates and N demands of phytoplankton; this allows more of the NH_4^+^_ flux to be oxidized during the day at deeper depths in the photic zone (Fig. 5C-D). A diel periodicity of this nature throughout much of the photic zone has important implications for estimates of new primary production, because it means that the bulk of regenerated NO_3^−^_ is produced at night. Uptake of this ‘regenerated’ NO_3^−^_ the following morning would be counted as new production, and result in an overestimation of carbon fluxes to the deep ocean.

## Conclusions

The results presented here are further evidence [24] that the WCA are a light-tolerant ecotype of AOA, able to remain active (Fig. 2) at high irradiance intensities (Fig. S1). Competition with phytoplankton for NH_4^+^_, rather than light itself, was the strongest regulator of WCA cellular activity (Fig. 2) and rates of nitrification (Fig. 1) during our experiments. Under a best-case scenario for AOA in surface waters of the ocean (ample substrate and low phytoplankton abundance in the ‘deep’ treatment) only a small fraction of the NH_4^+^_ pool was oxidized (≤17%) (Fig. 1B-C). Surprisingly, the AOA were unable to maintain a consistent rate of ammonia oxidation even at the lowest levels of phytoplankton growth (Fig. 1A-C), when no decline in their community size was apparent (Fig. 2). Within the ‘bloom’ treatment, intensifying competition with phytoplankton over the three day experimental period reduced nitrification rates from those typical of the base of the photic zone (∼60 nmol L^−1^ d^−1^) to those of well-lit layers (<1 nmol L^−1^ d^−1^) [5], [6], [21] (Fig. 1C).

Compared to the coastal ocean, phytoplankton in the oligotrophic ocean have adapted to the extremely low availability of NH_4^+^_ by maintaining a lower K_m_ and higher V_max_ for NH_4^+^_ [41], enabling them to capture small, episodic regenerative N fluxes [42]. Based on our results, it seems unlikely that the AOA are more adept at capturing NH_4^+^_ under these circumstances, consistent with decreasing rates of nitrification in the photic zone between coast and gyre [7]. Future work should address whether differences in kinetic traits of dominant phytoplankton could change the nature of this relationship, allowing for variability in nitrification rates in surface waters of the global ocean.

## Materials and Methods

Sample collection and experimentation were done aboard the R/V *Western Flyer* during October of 2011 at stations M1 (36.747 N, 122.022 W; bottom depth, 1000 m), M2 (36.751 N, 121.335 W; bottom depth, 1800 m) and 67–70 (37.281 N, 124.329 W; bottom depth,>3000 m) within and offshore of Monterey Bay (Northeast Pacific Ocean), as part of MBARI’s CANON initiative (cruise: CANON11). Seawater samples were collected using a previously described [43] profiling CTD-rosette system. Chlorophyll concentrations were determined at sea [43]. Samples for determination of NO_3^−^_, SiO_4^4−^_, PO_4^3−^_ and NO_2^−^_ concentrations were frozen at sea and transported to shore, where macronutrient concentrations were determined following previously described colorimetric methods [43]. NH_4^+^_ concentrations were measured in unfiltered seawater samples immediately after collection by fluorescence, as described previously [23].

### Multiday competition experiments

Waters used for multiday (competition) experiments were collected at station M1 (36.7470 N, 122.0220 W) from 2 m and 60 m. Upon return to deck, 2 m (‘surface’) and 60 m (‘deep’) waters were drained directly from the rosette into triplicate, acid rinsed 4 L polycarbonate bottles (3 per depth). To a third set of bottles, 0.4 L of 2 m water (10% of volume) were added to 3.6 L of 60 m water (90% of volume), to mimic mixing following an upwelling event (‘bloom’). The resultant 9 bottles (3 treatments×3 replicates) were subsampled for determination of chlorophyll, NO_3^−^_ and NH_4^+^_ concentrations, using the same methodologies described above. Due to the water constraints during the multiday experiments, chlorophyll concentrations were measured at sea on Days 0 and 3 and estimated for Days 1 and 2 assuming 1 µg L^−1^ chlorophyll accumulated for every 1 µmol L^−1^ of NO_3^−^_ depleted [44].

Following the initial subsampling, bottles from all treatments were placed into an on deck incubator cooled with ambient surface waters (12.5–16°C). The incubator was covered with neutral density screening, which attenuated incident irradiance by 50% (calibrated with a Biospherical QLS-100, San Diego, CA, USA). The intensity of irradiance reaching the deck incubator was monitored throughout our experiments using a photosynthetically active radiation (PAR) sensor (LI-COR, Lincoln, Nebraska, USA) affixed to the top of the incubator. The irradiance intensities during the experimental period, including start and end times, are shown in Figure S1.

Subsamples for determination of macronutrient and chlorophyll concentrations were taken on each day of the experiment, according to the methods described above. Additionally, an aliquot for water from each of the 4 L enclosures was transferred to clear 280 ml polycarbonate bottles for determination of nitrification rates. ^15^NH_4^+^_ tracer was added to each of the 280 ml incubation bottles (final ^15^N concentration  = 0.5 µmol L^−1^), after which the samples were mixed by inversion and placed in the on deck incubator for 24 hours. Subsamples (50 ml) for determination of the ^15^N enrichment of the NO_3^−^_+NO_2^−^_ pool, taken at 0 and 24 h, were passed through a 0.2 µm pore size syringe filter and stored frozen (−80°C) until analysis. At the end of the experiment, on Day 3, the water remaining in the 4 L incubation vessels (1 L) was filtered to harvest bacterioplankton biomass for later molecular genetic analyses (described below).

### Light manipulation experiments

Waters from 30 and 60 m at M1 and 30, 60 and 200 m at M2 were used to study the effects of light on nitrification in the absence of actively growing phytoplankton. For these experiments, water from each depth of study was drawn from the sampling rosette into either duplicate 280 ml polycarbonate (light) or 280 ml brown HDPE (dark bottles). Incubation experiments were initiated by addition of ^15^NH_4^+^_ tracer to a final concentration of 0.5 µmol L^−1^, after which bottles were held in the on deck incubator described above for 24 hours. Samples to determination the rate of ^15^N accumulation in the NO_3^−^_+NO_2^−^_ pool were taken at 0 and 24 hours, passed through a 0.2 µm pore size filter and frozen at −80°C.

### Diel periodicity experiments

Waters from five depths (2, 5, 10, 20 and 35 m) were collected before dawn at station 67–70 and drawn into clear 280 ml polycarbonate bottles (N = 2 per depth). Following addition of ^15^NH_4^+^_ tracer to a final concentration of 0.2 µmol L^−1^, seawater samples were incubated at estimated in situ levels of light (secchi disk according to Pennington et al. [43]), using stainless steel tubes pre-drilled with evenly spaced and sized holes, which were submerged in the deck incubator. Samples from depths 2, 5, 10, 20 and 35 m were incubated in light tubes transmitting 90, 50, 15 and 1 and 0.1% light, respectively, for 24 h. Samples to determine the rate of ^15^N accumulation in the NO_3^−^_+NO_2^−^_ pool were taken at 0, 8, 16 and 24 hours (Fig. 5), passed through a 0.2 µm pore size filter and frozen at −80°C until analysis.

### Stable isotope analysis and rate calculations

The δ^15^N of NO_3^−^_+NO_2^−^_ was determined at the University of California Davis Stable Isotope Facility (http://stableisotopefacility.ucdavis.edu/no3.html) using the denitrifier method [45], [46] and a ThermoFinnigan GasBench and PreCon trace gas concentration system interfaced to Delta V^PLUS^. δ ^15^N values were calibrated against nitrate isotope reference materials USGS32, USGS34 and USGS35 analyzed in parallel. Precision of the N isotope analysis, determined by repeat analysis of the reference materials, was found to be ±0.4 ‰ for the entire data set.

^15^N-ammonia oxidation rates (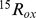) were determined on the basis of the accumulation of ^15^N in the NO_3^−^_+NO_2^−^_ pool relative to the initial enrichment of the NH_4^+^_ pool (atom % ^15^N)

using the equation developed by Ward [47],
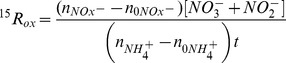
where 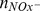 is the atom percent (at%) ^15^N in the NO_3^−^_+NO_2^−^_ pool measured at time (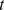), 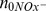 is the measured at% ^15^N of the NO_3^−^_+NO_2^−^_ pool at the start of the incubation, 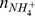 is the at% ^15^N enrichment of the NH_4^+^_ at the beginning of the experiment, and 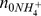 is the at% ^15^N enrichment of the ambient NH_4^+^_ pool, and 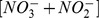 is the concentration of the NO_x_^−^ pool. For these calculations, the initial at% enrichment of the NH_4^+^_ pool (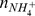) was calculated by isotope mass balance using NH_4^+^_ concentrations determined fluorometrically [48] at the time of sampling, assuming that the ^15^N activity of the ambient NH_4^+^_ pool (prior to tracer addition, 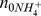) was 0.3663 at% ^15^N and that there was no significant dilution of the tracer pool over the course of our incubations [49].

### Cell harvesting and nucleic acids extraction

With the exception of seawater samples taken at the end of the multiday experiments, all samples were drawn directly from the sampling rosette into acid rinsed 1 L polycarbonate bottles and immediately filtered. Briefly, cells were harvested by pressure filtration of seawater samples (0.92 to 1 L for all experiments) through 25 mm filters housed in Swinnex filter holders (Millipore, Billerica, Massachusetts, USA); each sample was first passed through a 10 µm pore size polyester prefilter (GE Osmonics, Minnetonka, Minnesota, USA) and then a 0.2 µm filter (Supor, Pall Inc, Port Washington, New York, USA). Filters were then flash frozen in liquid nitrogen in gasketed 2 ml bead tubes containing a mixture of 0.1 and 0.5 mm glass beads.

Total nucleic acids were extracted from a single filter sample using a two-step co-extraction protocol, as described previously [23]. Briefly, the samples were first removed from storage at -80°C and immediately placed on ice. Then, 750 µl of lysis buffer (mirVana miRNA isolation kit (Life Technologies, Carlsbad, California, USA) was added to each tube, which was then sealed with parafilm and vortexed briefly to disperse the lysis buffer containing RNAse inhibitors. Following this, cell lysis was achieved by mechanical agitation in a FastPrep bead beater for two cycles of 45 seconds at setting 5.5. The tubes were then spun down to reduce foaming. The supernatant was passed through a DNeasy DNA capture column (Qiagen, Valencia, California, USA). Columns were then stored at 4°C until RNA extraction was completed (ca. 2 h). Column-bound DNA was purified and eluted using the DNeasy kit according to the manufacturer’s protocol, yielding an average (±S.D.) of 2.1±0.4 µg DNA L^−1^ of seawater.

Following passage through the DNeasy column, the eluent was immediately processed for total RNA purification using the mirVana miRNA isolation kit (Life Technologies, Carlsbad, California, USA) following the manufacturer’s protocol. RNA was eluted from the capture column with 75 µl of 95°C nuclease-free water. RNA yields averaged (±S.D.) 1.8±0.5 µg RNA L^−1^ of seawater. An aliquot of the purified RNA was immediately subjected to removal of contaminating DNA using the Turbo DNA-free kit following manufacturer’s protocol. cDNA was synthesized using random hexamers and the SuperScript III First-Strand Synthesis System for RT-PCR (Life Technologies, Carlsbad, California, USA) according to manufacturer’s protocol, except for increasing the reverse transcription incubation step to 5 hours at 50°C. Negative reverse transcription control reactions were performed for each sample, replacing the reverse transcriptase enzyme with water.

### Estimation of *amoA* gene and transcript abundances

The abundance of *amoA* genes related to WCA and WCB Thaumarchaeal ecotypes was estimated with two independent, non-overlapping qPCR assays [50]. For WCA the primers WCA-amoA-F (5’-ACACCAGTTTGGCTWCCDTCAGC-3’) and WCA-amoA-R (5’-TCAGCCACHGTGATCAAATTG-3’) and probe WCA-amoA-P (5’-FAM-ACTCCGCCGAACAGTATCA-BHQ1-3’) were used. For WCB the primers Arch-amoAFB (5’-CATCCRATGTGGATTCCATCDTG-3’) and WCA-amoAR (5’-AAYGCAGTTTCTAGYGGATC-3’) and probe WCA-amoA-P (FAM-CCAAAGAATATYAGCGARTG-BHQ1-3’) were used. The assays were run with identical qPCR reaction chemistries, as follows: 12.5 µL Taqman Environmental Master Mix 2.0 (Life Technologies, Carlsbad, CA, USA), 200 nM of each primer, 300 nM of each probe and either 1 µL of DNA or 2 µL of cDNA template per reaction (0.5 – 42 ng template DNA or cDNA per reaction), to a final volume of 25 µL. Cycling conditions were: 95°C for 10 min, 40 cycles of 95°C for 15 sec, 56°C for 1 min, followed by detection. The abundance of β-AOB *amoA* gene and transcript abundances were determined using commonly used oligonucleotides [32], [33] (amo1F/2R) and SYBR Green chemistry, as described previously [51]. Briefly, each 25 µl reaction contained: 12.5 µl Failsafe Green Real-Time PCR PreMix E (Epicentre Biotechnologies, Madison, WI, USA), 400 nM of each primer, 1.25 U of AmpliTaq LD (Life Technologies, Carlsbad, CA, USA) and ROX passive reference dye. The following thermal cycling profile was used for 35 cycles: 95°C for 45 s, 56°C for 30 s, 72°C for 50 s, and a plate reading step at 82°C for 10 s. Marine Group I archaea 16S rRNA and transcripts were amplified as described previously [52].

All qPCR assays were run in triplicate using a StepOnePlus Real-Time PCR System (Applied Biosystems). Standard curves ranging from 1 to 1×10^8^ gene copies per reaction were generated from purified, linearized plasmids obtained from clone libraries constructed with each primer set. The limit of detection of was 1 copy per reaction for WCA and WCB AOA *amoA*, and 10 copies per reaction for β-AOB *amoA* genes and transcripts. In the event that the coefficient of variation for a set of triplicate reactions exceeded 10%, one of the replicates was omitted or the sample was reanalyzed. Both assays had efficiencies of 94**–**99% across all samples; all results were consistent and reproducible. To facilitate comparison between samples, estimates of gene and transcript abundances obtained by qPCR were normalized to the volume of seawater filtered and reported with the units of genes L^−1^ or transcripts L^−1^ throughout the manuscript.

### Statistical analysis

All statistical analyses were performed on untransformed data in SPSS version 20 (IBM). Fit analysis for the predictive relationships was done with Igor Pro version 6.04 (Wavemetrics).

## Supporting Information

Figure S1 Flux of photosynthetically active radiation (PAR) to the deck incubator where all experiments were conducted. The yellow box highlights the incubation period for the light-dark experiments with waters from Station M1; the orange box denotes the incubation period for the same experiments conducted with waters from station M2 (Tables 1–2). The blue shaded region indicates the period of time over which the multiday experiments were performed aboard the R/V Western Flyer (Fig. 1–4). All experiments were conducted under neutral density screening that attenuated incident irradiance by 50%.
(TIF)

Table S1 Phytoplankton community structure assessed by epifluorescence microscopy and expressed as percent of total phytoplankton biomass as carbon, in surface waters (2 m) at the time of sampling stations M1 and M2.
(PDF)

Table S2 Abundance of AOA and β-AOB *amoA* and Thaumarchaeal 16S ribosomal RNA genes and transcripts at the start and end of multiday experiments. ^†^Values represent the average of replicate samples (N = 3 for ‘surface’, ‘bloom’; N = 2 for ‘deep’) for end point samples (± S.D). Initial time point samples were not replicated. B.D., below detection limit of assay (1 copy per qPCR reaction for WCA, WCB *amoA*; 10 copies per reaction for AOB *amoA*). All gene and transcript copies were normalized by the volume of seawater filtered for each sample, and expressed as copies per L^−1^ of seawater.
(PDF)
