# Supplementary figures and images for: Ammonium Uptake by Phytoplankton Regulates Nitrification in the Sunlit Ocean
Source: PLoS One. 2014 Sep 24;9(9):e108173. doi: 10.1371/journal.pone.0108173 (PMC4177112; doi:10.1371/journal.pone.0108173)

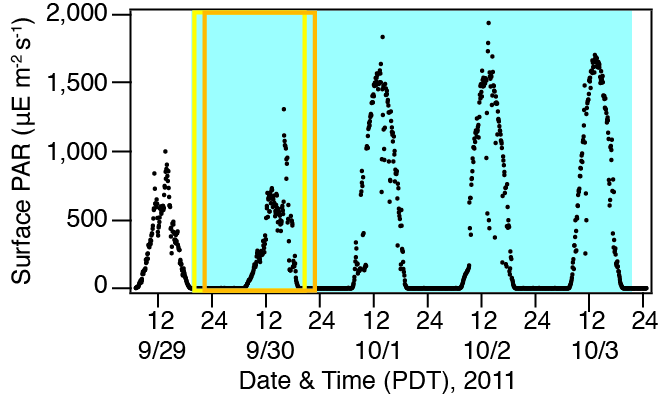

Supplement: Figure S1 — Flux of photosynthetically active radiation (PAR) to the deck incubator where all experiments were conducted. The yellow box highlights the incubation period for the light-dark experiments with waters from Station M1; the orange box denotes the incubation period for the same experiments conducted with waters from station M2 (Tables 1–2). The blue shaded region indicates the period of time over which the multiday experiments were performed aboard the R/V Western Flyer (Fig. 1–4). All experiments were conducted under neutral density screening that attenuated incident irradiance by 50%. (TIF) [file pone.0108173.s001.tif]
