# Supplementary material for: Ammonium Uptake by Phytoplankton Regulates Nitrification in the Sunlit Ocean
Source: PLoS One. 2014 Sep 24;9(9):e108173. doi: 10.1371/journal.pone.0108173 (PMC4177112; doi:10.1371/journal.pone.0108173)
Supplement: Table S1 — Phytoplankton community structure assessed by epifluorescence microscopy and expressed as percent of total phytoplankton biomass as carbon, in surface waters (2 m) at the time of sampling stations M1 and M2. (PDF) [file pone.0108173.s002.pdf]

Supporting Information:

Table S1. Phytoplankton community structure assessed by epifluorescence microscopy and expressed as percent of total phytoplankton biomass, in surface waters (2 m) at the time of sampling MBTS stations M1 and M2 in September 2011.

Phytoplankton Group	M1	M2
Synechococcus	6.4%	19.1%
Red Fluorescing Picoplankton	1.7%	11.1%
Prymnesiophyte	6.4%	5.5%
Autotrophic Flagellate	0.6%	0.0%
Autotrophic Dinoflagellate	26.7%	16.3%
Cryptophyte	5.9%	4.1%
Prasyinophyte	0.6%	0.2%
Phaeophyte	2.4%	0.0%
Pennate Diatoms	7.7%	2.5%
Centric Diatoms	41.6%	41.2%
