# Supplementary material for: Ammonium Uptake by Phytoplankton Regulates Nitrification in the Sunlit Ocean
Source: PLoS One. 2014 Sep 24;9(9):e108173. doi: 10.1371/journal.pone.0108173 (PMC4177112; doi:10.1371/journal.pone.0108173)
Supplement: Table S2 — Abundance of AOA and β-AOB amoA and Thaumarchaeal 16S ribosomal RNA genes and transcripts at the start and end of multiday experiments. †Values represent the average of replicate samples (N = 3 for ‘surface’, ‘bloom’; N = 2 for ‘deep’) for end point samples (± S.D). Initial time point samples were not replicated. B.D., below detection limit of assay (1 copy per qPCR reaction for WCA, WCB amoA; 10 copies per reaction for AOB amoA). All gene and transcript copies were normalized by the volume of seawater filtered for each sample, and expressed as copies per L−1 of seawater. (PDF) [file pone.0108173.s003.pdf]

Table S2. Abundance of AOA and AOB *amoA* and Thaumarchaeal 16S ribosomal RNA genes and transcripts at the start and end of multiday experiments.

	WCB *amoA* genes L^{-1}		WCB *amoA* mRNA L^{-1}		β-AOB *amoA* genes L^{-1}		β-AOB *amoA* mRNA L^{-1}		MG1 16S genes L^{-1}		MG1 16S transcripts L^{-1}		WCA *amoA* genes L^{-1}		WCA amoA mRNA L^{-1}	
Day	0	3	0	3	0	3	0	3	0	3	0	3	0	3	0	3
Surface†	$4x10^{3}$	$1x10^{2}$ $\pm2x10^{1}$	BD	BD	$2x10^{4}$	$9x10^{3}$ $\pm2x10^{3}$	BD	BD	$3x10^{5}$	$1x10^{5}$ $\pm1x10^{4}$	$2x10^{4}$	$9x10^{2}$ $\pm3x10^{2}$	$7x10^{5}$	$2x10^{5}$ $\pm9x10^{4}$	$3x10^{3}$	$5x10^{2}$ $\pm2x10^{2}$
Deep	$2x10^{5}$	$1x10^{5}$ $\pm4x10^{4}$	$4x10^{3}$	BD	$3x10^{5}$	$2x10^{5}$ $\pm3x10^{4}$	$5x10^{3}$	BD	$5x10^{6}$	$3x10^{6}$ $\pm7x10^{5}$	$8x10^{6}$	$3x10^{6}$ $\pm1x10^{6}$	$4x10^{6}$	$4x10^{6}$ $\pm3x10^{5}$	$4x10^{5}$	$7x10^{4}$ $\pm2x10^{4}$
Bloom	$2x10^{5}$	$2x10^{4}$ $\pm1x10^{3}$	$7x10^{3}$	BD	$4x10^{5}$	$7x10^{4}$ $\pm2x10^{4}$	$7x10^{3}$	BD	$4x10^{6}$	$2x10^{6}$ $\pm3x10^{5}$	$6x10^{6}$	$2x10^{5}$ $\pm2x10^{4}$	$4x10^{6}$	$2x10^{6}$ $\pm2x10^{5}$	$5x10^{6}$	$1x10^{4}$ $\pm4x10^{1}$

†Values represent the average of replicate samples (N=3 for 'surface', 'bloom'; N=2 for 'deep') for end point samples (± S.D). Initial time point samples were not replicated. BD, below detection limit of assay (1 copy per qPCR reaction for WCA, WCB; 10 copies per reaction for AOB).
